# Acute Short-Term Mental Stress Does Not Influence Salivary Flow Rate Dynamics

**DOI:** 10.1371/journal.pone.0051323

**Published:** 2012-12-13

**Authors:** Ella A. Naumova, Tudor Sandulescu, Philipp Al Khatib, Michael Thie, Wing-Kee Lee, Stefan Zimmer, Wolfgang H. Arnold

**Affiliations:** 1 Witten/Herdecke University, Faculty of Health, School of Dentistry, Witten, Germany; 2 University of Duisburg-Essen, Institute of Pathology and Neuropathology, Essen, Germany; 3 Witten/Herdecke University, Institute for Physiology & Pathophysiology, Centre for Biomedical Education and Research, Witten, Germany; Max Planck Institute of Psychiatry, Germany

## Abstract

Background: Results of studies that address the influence of stress on salivary flow rate and composition are controversial. The aim of this study was to reveal the influence of stress vulnerability and different phases of stress reactivity on the unstimulated and stimulated salivary flow rate. We examined that acute mental stress does not change the salivary flow rate. In addition, we also examined the salivary cortisol and protein level in relation to acute mental stress stimuli. Methods: Saliva of male subjects was collected for five minutes before, immediately, 10, 30 and 120 min after toothbrushing. Before toothbrushing, the subjects were exposed to acute stress in the form of a 2 min public speech. Salivary flow rate and total protein was measured. The physiological stress marker cortisol was analyzed using enzyme-linked immunosorbent assay. To determine the subjects' psychological stress reaction, the State-Trait-Anxiety Inventory State questionnaire (STAI) data were obtained. The subjects were divided into stress subgroup (S1) (psychological reactivity), stress subgroup (S2) (psychological and physiological reactivity) and a control group. The area under the curve for salivarycortisol concentration and STAI-State scores were calculated. All data underwent statistical analysis using one-way analysis of variance. Results: Immediately after stress exposure, all participants exhibited a psychological stress reaction. Stress exposure did not change the salivary flow rate. Only 69% of the subjects continued to display a physiological stress reaction 20 minutes after the public talk. There was no significant change in the salivary flow rate during the psychological and the physiological stress reaction phases relative to the baseline. Conclusions: Acute stress has no impact on the salivary flow rate; however, there may be other responses through salivary proteins that are increased with the acute stress stimuli. Future studies are needed to examine specific proteins and their possible roles in acute stress responses.

## Introduction

The salivary flow rate influences the chemical environment [1], the demineralization/remineralization process [2] and it provides a cleaning effect for salivary clearance in the oral cavity [3], [4], [5]. The salivary flow rate is used to aid in the diagnosis of oral and systemic diseases [6]. For these purposes, it is important to understand the different aspects of saliva secretion in healthy people, including its kinetics and the influencing factors.

The salivary flow rate has a specific dynamics that depends on the circadian rhythm [7], age [1], body mass index (BMI) [7] and climate [8]. The saliva flow rate can be stimulated by mechanical, gustatory, olfactory and psychological factors [2].

Saliva secretion is regulated by the autonomic nervous system [1], [9], [10]. Rohleder et al. [11], discussed the concurrent inhibition of the parasympathetic nervous system during acute mental stress exposure as a cofounder of salivary flow rate suppression. A number of studies have focused on the evaluation of the salivary secretion in healthy people during stress responses to acute mental stress [2], [12], [13]. However, the data on the influence of mental stress on the salivary flow rate have not indicated the presence of a specific influence [12], increased salivary flow rate [2] or reduced salivary flow rate [6].

Stress symptoms are induced by the autonomic nervous system [14] and the reactions of the hypothalamic-pituitary-adrenal axis (HPA) [15]. Acute mental stress in healthy people induces such phenomena as a feeling of dry mouth, adhesion of the tongue to the palate and swallowing difficulties [6], [16]. The feeling of dry mouth is commonly thought to be caused by reduced salivary secretion.

Thus, different types of vulnerability exhibit different mechanisms of autonomic and HPA activation and trigger different stress reactions on a psychological or on both psychological and physiological levels. Bjorntorp et al. [17] studied the cascade of events along the stress axes. Balodis et al. [18] reported on stress dynamics and subsequent stress reactivity. Boudarene et al. [19] proposed a gradual interpretation of stress reaction by distinguishing between primary and secondary protest. As a result of two phases of stress reactions (the primary protest and the subsequent secondary protest), Boudarene et al. [19] identified three types of stress responses with three respective types of vulnerability: 1) without anxiety (psychological silence) and without an increase in cortisol level (biological silence), indicating less vulnerability; 2) high level of anxiety (psychological manifestations) but no increase in cortisol level (biological silence), indicating psychological vulnerability; and 3) high state of anxiety and increased plasma cortisol levels (psychological and physiological manifestations), indicating psychological and physiological vulnerability.

The stress reactions at the beginning of the stressful event (primary protest) reflect the activation of the sympathetic nerve system (SNS) and can be monitored by measuring alpha-amylase levels [10], [11], [20], [21]. Subsequent reactions (secondary protest) reflect the activation of the HPA, and salivary cortisol levels can be used as a marker [12], [19], [21].

Controversial results have been reported about the influence of acute mental stress on the salivary flow rate in terms of salivary secretion and stress vulnerability types or phases of stress reactivity [3], [9], [11], [14], [21], [22], [23], [24], [25], [26]. Therefore, the aim of this research was to investigate the salivary flow rate dynamics during an experimental acute mental stress situation in subjects with different stress vulnerability types and during the phases of stress reactivity. The following null hypothesis was proposed: the different stress vulnerability types and phases of stress reactivity have no influence on the salivary flow rate after acute mental stress.

## Materials and Methods

### Subjects

Sixty-four healthy male dental and medical students in their first, second and third years of study (mean age: 23.83±2.34 years; body weight: 79.30±7.29 kg) were recruited and randomized in this parallel study. Female subjects were excluded to avoid influences of the female hormonal cycle on cortisol measurements [24], [27]. After receiving verbal and written information on the investigation, the subjects provided their informed consent. All subjects received written instructions regarding the schedule of the study design and the proper toothbrushing method. Inclusion criteria were satisfactory oral and general health. Exclusion criteria were hormonal medicaments and the presence of endocrine disease. The test subjects were divided into two groups the control (C; n = 32) and stress (S; n = 32) groups. After cortisol levels were determined, the stress group was divided into two subgroups: the subgroup that exhibited only a psychological stress reaction (S1; n = 10) and the subgroup that exhibited both psychological and physiological stress reactions (S2; n = 22).

### Study design

This protocol was approved by the ethical committee of Witten/Herdecke University (permission 39/2009). The study subjects' self-reported psychological health states and baseline anxiety levels were determined by the answers provided on the Hospitality Anxiety and Depression Scale (HADS), [28], [29], and the State-Trait-Anxiety Inventory (STAI-Trait) [30] at home in a silent atmosphere.

**Figure 1:**
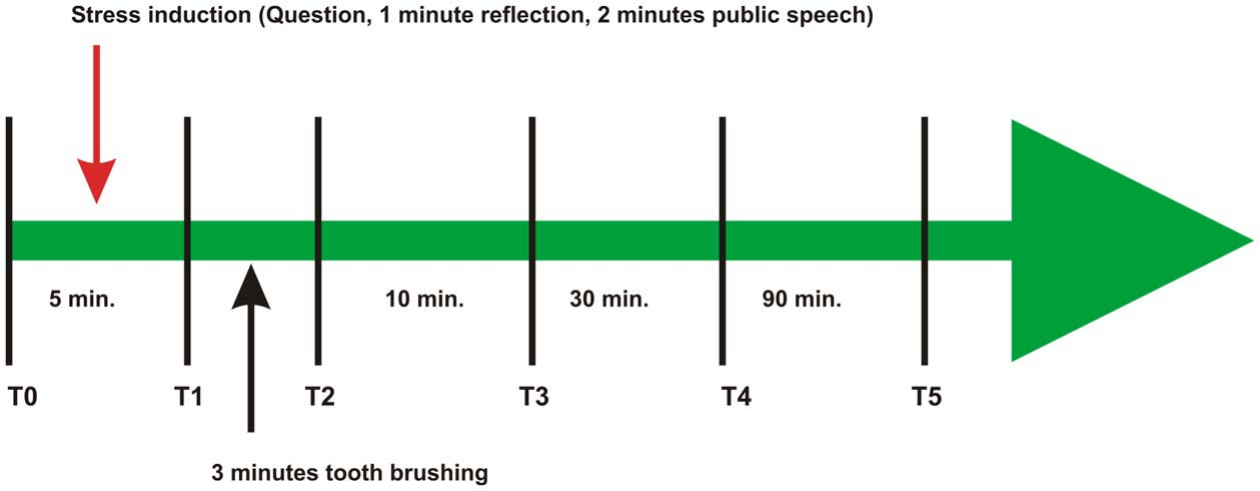
Graphic summary of the experimental procedure.

To avoid influences of the circadian rhythm on cortisol concentration, all saliva samples were collected between 2:00 and 4:30 p.m. from September to November [22], [31]. All subjects were asked not to eat or chew chewing gum at least one hour before the test and refrain from eating during the entire test period. At 2:00 p.m., unstimulated saliva was collected for five minutes as baseline samples (T0). Next the test subjects were exposed to acute stress by presenting a public talk for two minutes [32]. A representative from the audience, which included university lecturers and students, informed the subject immediately after he entered the classroom that he would receive a general-knowledge question that was not related to his educational specialization and that the speech will be recorded and evaluated. After one minute of reflection time, the subjects began their two-minute speech. Unstimulated saliva samples were collected immediately after the public talk (T1). To obtain stimulated saliva, all subjects were asked to brush their teeth for three minutes with a commercially available toothpaste. Immediately (T2), samples of stimulated saliva and of 10 (T3), 30 (T4) and 120 (T5) minutes post-toothbrushing unstimulated saliva were collected. The whole experimental procedure is shown in Fig. 1. The control groups underwent the same chronological protocol but without presenting the public talk. To measure the self-reported psychological stress reactions, the subjects answered the STAI-State questionnaires at the same time as saliva sampling [30].

### Psychological health evaluation

The HADS consists of 14 items that address various aspects of depression and anxiety over the most recent seven days. This test has been used to identify subjects with depression which were excluded from the study. The scale can be divided into two subscales (anxiety and depression), with higher scores indicating more anxiety and more depression, respectively. Sum scores <8 indicate the normal range, scores of 8–10 reflect mild alterations, and scores ≥11 indicate clinically relevant symptoms [28], [29].

### Toothbrushing and saliva sampling

A total of six saliva samples were collected from each test subject (T0 through T5). For standardization, all subjects brushed their teeth with commercially available toothpaste using the same brushing technique [33] for three minutes. Saliva samples were collected into 20 ml tubes. During saliva sampling, all subjects were seated in a silent atmosphere with their eyes open and head tilted slightly forward. All saliva samples were without visible blood contamination [15]. Immediately after collection, the saliva's weight was determined. 100 µl of saliva was frozen and stored at −80°C until it was assayed for total protein content. The rest of the sample was centrifuged (B Centrifuge, Beckman Instruments Inc., Germany) for 10 min at 3000× g. An aliquot was taken, frozen and stored at −80°C until it was assayed for cortisol concentration.

### Psychological and physiological stress reaction phase evaluation

The validated State-Trait-Anxiety Inventory questionnaire was used to assess the baseline anxiety (STAI-Trait) and the psychological response to the stress resulting from the public talk (STAI-State) [30], [32], [34]. Each of the STAI subscales (i.e., STAI-State and STAI-Trait) contains 20 items that address worry, tension, apprehension, and nervousness. Subscale scores range from 20 to 80, with higher scores indicating increased anxiety. The psychological stress reaction was represented by increased STAI-State values after stress exposure relative to baseline.

Salivary cortisol levels were measured using a commercial enzyme-linked immunosorbent assay (Cortisol ELISA, IBL International, Hamburg, Germany) according to the manufacturer's instructions. The cross-reactivity of the anti-cortisol antibody with other relevant steroids was 7.0% for 11deoxycortisol, 4.2% for cortisone, 1.4% for corticosterone, 0.35% for progesterone, and <0.01% for testosterone, estrone, estradiol, and estriol. The intra- and interassay variances were 4.8% and 5.9%, respectively. Physiological stress reactions were represented by increased cortisol concentration after the stress exposure relative to baseline.

### Amount of saliva and salivary secretion rate

The weight of the saliva (grams) was determined using the electronic Micro-, Analysis and Precision Scale Sartorius (CP Series CPA 1245, Göttingen, Germany). To calculate the salivary secretion rate (ml/min) for unstimulated saliva, the quantities of the saliva samples (T0, T1, T3, T4 and T5) were divided by five; the quantities for stimulated saliva (T2) were divided by three. Saliva is composed of more than 99% water [2], therefore, the conversion from grams to milliliters was one to one.

### Total salivary protein measurements

100 µl saliva was centrifuged at 3000× g. Total protein contents was photometrical measured using Coomassie® Brilliant Blue 250 (Bio-Rad Laboratories GmbH, Munich, Germany) at 595 nm. As standards served 0.1, 0.3, 0.5 and 0.7 mg/ml solutions of albumin [35].

### Statistical methods

The obtained data were processed with the Statistical Package for Social Sciences (SPSS 20.0, Chicago, III, USA). Prior to the experiments a power analysis was performed with a power of 0.8 and a significance level of α<0,05 which revealed a minimum number of 13 subjects. Therefore 16 test subjects were selected for each group. The STAI-state score, the salivary cortisol concentration and the salivary flow rate were analyzed at each collection point (T0–T5). Additionally the area under the curve (AUC) was calculated and compared using one-way analysis of variance (one-way ANOVA) and post-hoc Bonferroni adjustment. The protein concentrations were compared with the non-parametric Wilcoxon-Mann-Whitney test. The correlation analysis between salivary flow rate, STAI-State scores and salivary cortisol concentration for each group (control, S1, S2) was performed by the Spearman Rho correlation test with a significance level p<0.01. The influence of repeated administration of the STAI questionnaire was tested with multivariance analyses for repeated measurements.

## Results

### Participant characteristics

No subjects were excluded because of a state of anxiety or depression (HADS score <7).

### Psychological and physiological reactions after the public talk stressor

The stress subgroup that exhibited only a psychological stress reaction (S1, n = 10) had increased STAI-State scores and no increase in the salivary cortisol concentration, while the subgroup with both psychological and physiological stress reactions (S2, n = 22) had increased STAI-State scores and increased salivary cortisol concentrations (ANOVA group interaction effect for AUC STAI-State scores over T0 to T5 F = 13.91; p<0.001; df = 2 and for AUC cortisol concentration over T0 to T5 F = 21.85; p<0.001; df = 2).

**Figure 2:**
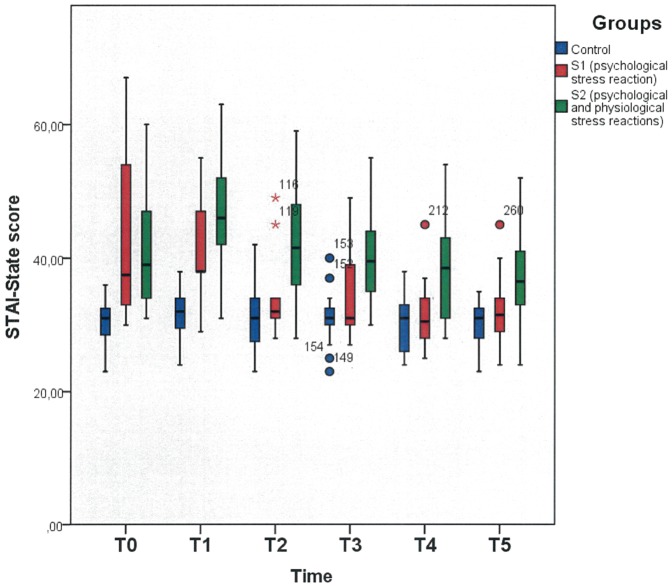
STAI-State scores of the participants in the three groups the control (C) and both stress subgroups (S1 and S2) before (T0) and immediately after presenting a public talk (T1) and immediately (T2), 10 (T3), 30 (T4) and 120 (T5) minutes after toothbrushing. Higher STAI-State scores indicate an elevated anxiety state in both stress subgroups (S1 and S2) compared to the control group (AUC STAI-state T0–T5: F = 13.91; p<0.001 ANOVA group interaction). The box indicates 50% of all measured values. The horizontal line within the box indicates the median. The whiskers below and on top of the box indicate the 75th percentile. Outliers are marked with circles.

STAI-State scores were significantly increased across the T0, T1, T2, T3, T4 and T5 collection points in both stress subgroups (S1 and S2) compared with the control group (ANOVA group interaction effect for T0: F = 9.73; p<0.001; df = 2; T1: F = 22.22; p<0.001; df = 2; T2: F = 11.57; p<0.001; df = 2; T3: F = 10.27; p<0.001; df = 2; T4: F = 8.60; p = 0.001; df = 2; T5: F = 5.797; p = 0.006; df = 2) Fig. 2 shows the increased STAI values even 120 minutes after stress exposure.

**Figure 3:**
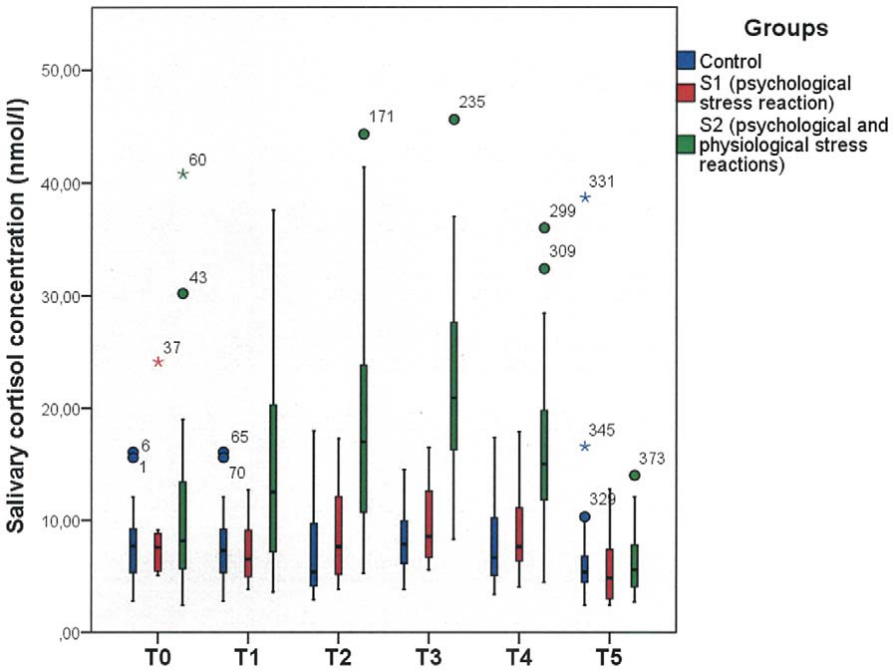
Dynamics of the salivary cortisol concentration (nmol/l) over a two-hour period (T0–T5) in the control group and both stress subgroups. Group S1 showed no significant increase in salivary cortisol concentration. Group S2 showed a significant increase in salivary cortisol concentration at T1, T2, T3 and T4 compared to the control group (T0: F = 2.01; p = 0.142; T1: F = 10.23; p<0.001; T2: F = 19.77; p<0.001; T3: F = 42.15; p<0.001; T4: F = 19.16; p<0.001; T5: F = 0.189; p = 0.829 ANOVA group interaction). For further description, see Fig. 1.

Presenting a public talk induced a significant increase in the salivary cortisol concentration in the stress subgroup S2 (n = 22, approximately 69% of the stress group) across the time points T1, T2, T3 and T4, while no changes were observed in the stress subgroup S1 (n = 10, approximately 31% of the stress group) relative to the control group (ANOVA group interaction effect for T0: F = 2.01; p = 0.142; df = 2; T1: F = 10.23; p<0.001; df = 2; T2: F = 19.77; p<0.001; df = 2; T3: F = 42.15; p<0.001; df = 2; T4: F = 19.16; p<0.001; df = 2; T5: F = 0.189; p = 0.829; df = 2) Fig. 3 demonstrates the increased cortisol levels after stress exposure in the group with psychological and physiological stress reactions. No differences can be seen between the control groups and the psychological stress group (S1).

Baseline cortisol levels over all test groups were 9.05±6.28 nmol/l. The ANOVA group differentiation between control, S1 and S2 groups revealed no group differences (F = 2.01; p = 0.142 ANOVA group interaction).The physiological stress reaction returned to the baseline cortisol level two hours after stress exposure (cortisol concentration T5: 6.55±5.02 nmol/l; F = 1.89; p = 0.829 ANOVA group interaction) while psychological stress reaction remained significantly elevated for up to two hours after stress exposure (STAI-State score: T0: C: 30.56±3.82; S1: 42.90±12.79; S2: 41.18±8.23; F = 9.73; p<0.001; df = 2 and T5: C: 30.25±3.62; S1: 32.10±6.54; S2: 37.32±8.07; F = 5.79; p = 0.006; df = 2; ANOVA group interaction). The correlation analysis between the STAI-State score and salivary cortisol concentration during T0 to T5 revealed no significant correlation in all groups. The results are summarized in Tab. 1. An influence of repeated application of the STAI questionnaire could be eliminated because the inter subject effects between the tests were significantly different (p<0.001).

**Table 1:** Correlation between STAI-State score and salivary cortisol concentration.

	control			S1			S2		
	r	p	df	r	p	df	r	p	df
T0	−0.021	0.939	16	−0.48	0.16	10	0.3	0.174	22
T1	−0.127	0.639	16	0.15	0.679	10	−0.43	0.85	22
T2	0.124	0.647	16	−0.122	0.736	10	−0.158	0.483	22
T3	−0.184	0.494	16	0.329	0.353	10	−0.071	0.754	22
T4	−0.441	0.87	16	0.055	0.881	10	−0.241	0.28	22
T5	−0.075	0.783	16	0.15	0.679	10	−0.237	0.289	22

### Amount of saliva

**Figure 4:**
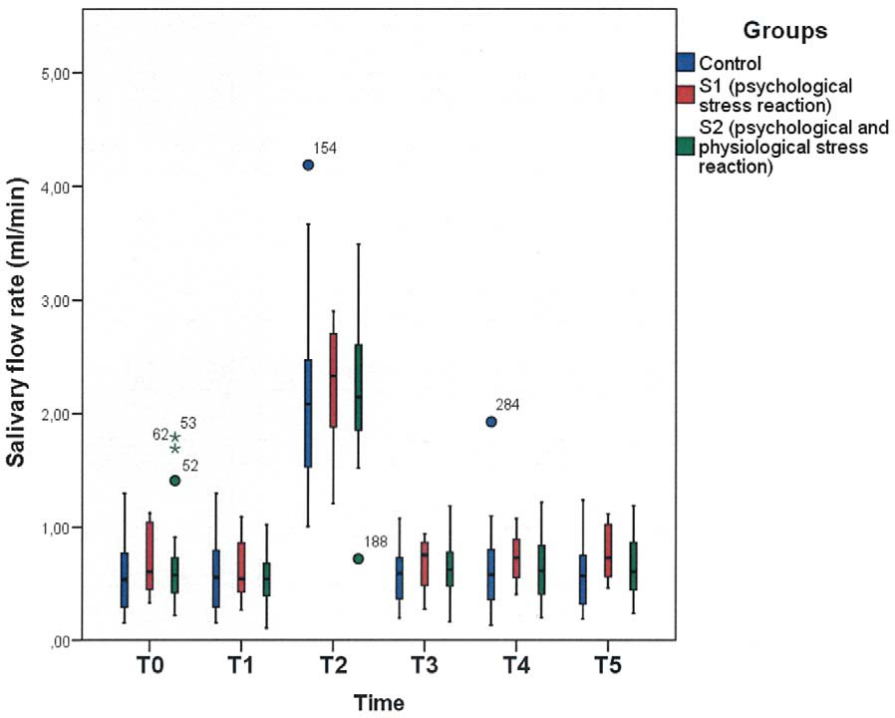
The dynamics of saliva secretion rate (ml/min) over a two-hour period (T0–T5) in the control group (C) and the stress subgroups (S1 and S2). There was no significant difference between the control group (C) and the two stress subgroups (T0: F = 0.91; p = 0.408; T1: F = 0.175; p = 0.840; T2: F = 0.28; p = 0.752; T3: F = 1.13; p = 0.329; T4: F = 0.62; p = 0.539; T5: F = 1.89; p = 0.160; AUC over T0 to T5 time interval excluding T2: F = 0.372; p = 0.544; ANOVA group interaction). For further description, see Fig. 1.

In the control group, the baseline value (T0) of unstimulated saliva was 0.5716±0.3149 ml/min and showed no significant difference to the stress group values (0.6907±0.3930 ml/min; p = 0.186; F = 1.78 ANOVA group interaction effect). The unstimulated salivary flow rates for the control and the respective stress groups (S1 and S2) showed no significant differences among the T1, T3, T4 and T5 collection points (T0: F = 0.91; p = 0.408; df = 2; T1: F = 0.175; p = 0.840; df = 2; T3: F = 1.13; p = 0.329; df = 2; T4: F = 0.62; p = 0.539; df = 2; T5: F = 1.89; p = 0.160; df = 2; AUC over T0 to T5 time interval excluding T2: F = 0.372; p = 0.544; df = 1; ANOVA group interaction) Fig. 4 shows the increase in the salivary flow rate at T2 after tooth brushing..

The stimulated salivary flow rate (T2) in the stress group (S1 and S2) showed no significant difference relative to the control group (p = 0.752; F = 0.28; df = 2; ANOVA group interaction).

The correlation analysis between the salivary flow rate and the STAI-Sate score and salivary cortisol concentration, respectively, showed no significant correlations. These results are summarized in Tables 2 and 3.

**Table 2:** Correlation between STAI-State score and salivary flow rate.

	control			S1			S2		
	r	p	df	r	p	df	r	p	df
T0	−0.224	0.405	16	0.018	0.96	10	−0.11	0.626	22
T1	-0.198	0.462	16	−0.106	0.77	10	−0.036	0.873	22
T2	−0.099	0.716	16	0.073	0.840	10	−0.213	0.341	22
T3	0.136	0.616	16	0.079	0.828	10	−0.1	0.657	22
T4	−0.105	0.699	16	0.345	0.328	10	0.012	0.958	22
T5	−0.169	0.532	16	−0.567	0.087	10	−0.308	0.163	22

**Table 3:** Correlation between salivary flow rate and cortisol concentration.

	control			S1			S2		
	r	p	df	r	p	df	r	p	df
T0	−0.157	0.389	32	0.188	0.602	10	−0.112	0.619	22
T1	0.096	0.6	32	−0.03	0.934	10	−0.023	0.92	22
T2	0.182	0.318	32	−0.091	0.803	10	−0.118	0.601	22
T3	0.224	0.217	32	−0.079	0.829	10	0.179	0.425	22
T4	0.267	0.14	32	−0.224	0.533	10	0.077	0.734	22
T5	0.217	0.233	32	0.171	0.637	10	0.193	0.390	22

### Total salivary protein content

**Figure 5:**
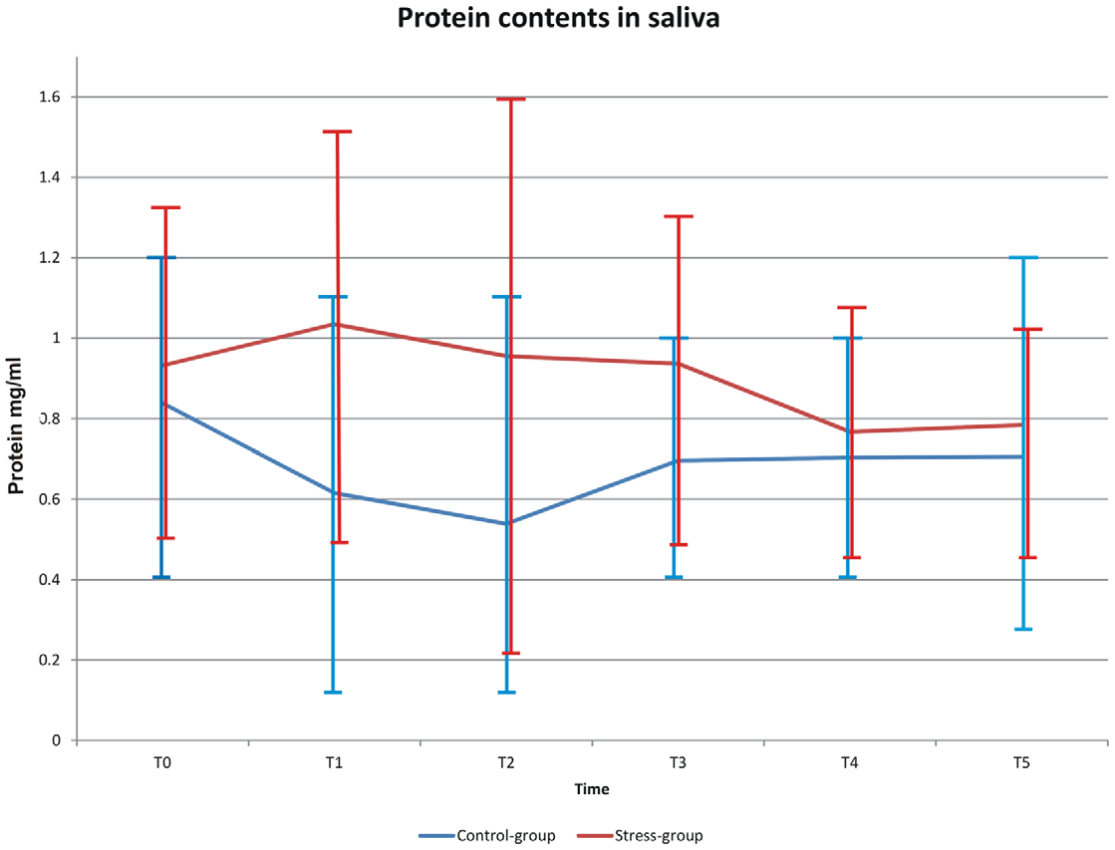
Dynamics of the protein concentration (mg/ml) in saliva in the control and stress groups.

At baseline (T0) the protein content in the saliva of both groups was similar (0.83 mg/ml control and 0.93 mg/ml stress group). At T1 immediately after stress exposure in the experimental group the protein content increased up to 1.03 mg/ml whereas in the control group no increase could be measured (0.61 mg/ml). The protein content in the stress group decreased again at T4 and was similar to that of the control group (Fig. 5).The difference in the protein contents was significant at T1, T2 and T3 (p<0.05).

## Discussion

In the present study, we proposed a differentiated approach to the investigation of salivary flow rate after acute mental stress by distinguishing among the different phases of stress reactivity. A psychological stress reaction (increasing STAI-Sate score) can be distinguished from a physiological stress reaction (activation of the HPA-axis with an increase of plasma and salivary cortisol). From the literature, it is known that there are various stress reaction phases [18], [19]. Two phases of stress reactions were identified in this study (namely, psychological and psychological). It is also known that certain subjects do not respond to acute short-term stress with the activation of the HPA-axis and that stress reactions are highly variable on an individual basis [18], [36].

The application of two different methods for stress verification (the STAI-State questionnaire and salivary cortisol level analyses) helped to determine the two phases of the stress reactivity (psychological and physiological reactions), which occurred consecutively. This finding was consistent with studies that reported the cascade of events along the stress axes [17] and suggested a gradual interpretation of the stress reaction by distinguishing between primary and secondary protests [19] and the subsequent stress reactivity [18].

The evaluation of the two phases of stress reactivity demonstrated that they have different starting times (the psychological phase started immediately, while the physiological phase occurred 20 minutes later) and different peak times (because the physiological phase has a 20-minute latency), but both phases returned to baseline levels two hours after stress induction. These results are in accordance with those of previous research, which indicated that the latency time between the removal of a stress reaction and the decrease of the blood cortisol level is, on average 15 to 20 minutes [23].

However, it has been shown in this study that the gradual nature of the psychological and physiological phases of stress reactivity has no impact on the flow rate dynamics of the stimulated and unstimulated saliva after acute short-term mental stress.

In the present study, individual differences in human stress vulnerability were also analyzed. Immediately after their exposure to short-term mental stress, all of the test subjects presented increased state anxiety scores (primary protest or psychological stress reactions). In approximately 31% of test subjects (S1), only psychological stress reactions were observed; 20 min later, 69% of the test subjects (S2) demonstrated secondary protest (physiological stress reactions). These findings are consistent with those of a previous study [36].

There was no correlation between psychological and physiological stress reactions (a pronounced psychological stress component was not associated with an increased physiological stress component), which is in agreement with the data of Boudarene et al. [19]. The variation of the flow rate of stimulated and unstimulated saliva did not correlate with the data from the STAI-State questionnaire score and the salivary cortisol levels for different stress vulnerability types.

A novel finding of this study was that the salivary flow rate dynamics were not altered during the psychological and the physiological phases of stress reactions after short-term mental stress. However, salivary protein concentration increased after stress exposure indicating a change in the composition of saliva. These data were consistent with previous studies [12]. Further, more detailed studies are needed to elaborate a possible change of salivary protein composition

These findings lead to the assumption that the main cause of the feeling of dry-mouth after acute short-term mental stress is not a change in the salivary flow rate but changes in the salivary composition.

A limitation of this study is that it does not take into account chronic stress which could also have an effect on salivary composition. Another limitation is that only male subjects could be included into this study because of the influence of the female hormonal cycle on the cortisol measurements. Therefore, the conclusions cannot be generalized.

The combination of the self-reported stress analysis and salivary cortisol measurement provides new opportunities for stress estimation in both investigatory experimental studies and daily medical practice, such as the further investigation of dry-mouth syndrome.
